# Treg cells-derived exosomes promote blood-spinal cord barrier repair and motor function recovery after spinal cord injury by delivering miR-2861

**DOI:** 10.1186/s12951-023-02089-6

**Published:** 2023-10-04

**Authors:** Guang Kong, Wu Xiong, Cong Li, Chenyu Xiao, Siming Wang, Wenbo Li, Xiangjun Chen, Juan Wang, Sheng Chen, Yongjie Zhang, Jun Gu, Jin Fan, Zhengshuai Jin

**Affiliations:** 1 The Affiliated Jiangsu Shengze Hospital of Nanjing Medical University, Suzhou, Jiangsu China; 2 The First Affiliated Hospital of Nanjing Medical University, Nanjing, Jiangsu China; 3 Nanjing Medical University, Nanjing, Jiangsu China; 4 Department of human anatomy, School of Basic Medicine, Nanjing Medical University, Nanjing, Jiangsu China

**Keywords:** Spinal cord injury, Regulatory T cells, Blood-spinal cord barrier, Exosomes, miRNA

## Abstract

The blood-spinal cord barrier (BSCB) is a physical barrier between the blood and the spinal cord parenchyma. Current evidence suggests that the disruption of BSCB integrity after spinal cord injury can lead to secondary injuries such as spinal cord edema and excessive inflammatory response. Regulatory T (Treg) cells are effective anti-inflammatory cells that can inhibit neuroinflammation after spinal cord injury, and their infiltration after spinal cord injury exhibits the same temporal and spatial characteristics as the automatic repair of BSCB. However, few studies have assessed the relationship between Treg cells and spinal cord injury, emphasizing BSCB integrity. This study explored whether Treg affects the recovery of BSCB after SCI and the underlying mechanism. We confirmed that spinal cord angiogenesis and Treg cell infiltration occurred simultaneously after SCI. Furthermore, we observed significant effects on BSCB repair and motor function in mice by Treg cell knockout and overexpression. Subsequently, we demonstrated the presence and function of exosomes in vitro. In addition, we found that Treg cell-derived exosomes encapsulated miR-2861, and miR-2861 regulated the expression of vascular tight junction (TJs) proteins. The luciferase reporter assay confirmed the negative regulation of IRAK1 by miR-2861, and a series of rescue experiments validated the biological function of IRAKI in regulating BSCB. In summary, we demonstrated that Treg cell-derived exosomes could package and deliver miR-2861 and regulate the expression of IRAK1 to affect BSCB integrity and motor function after SCI in mice, which provides novel insights for functional repair and limiting inflammation after SCI.

**Supplementary Information:**

The online version contains supplementary material available at 10.1186/s12951-023-02089-6.

## Introduction

Spinal cord injury (SCI) often occurs after traumatic injuries such as traffic accidents and falls from height. It is a destructive neurological and pathological state that can lead to motor, sensory, and autonomic dysfunctions, bringing great physical and psychological pain to patients. The prevalence of spinal cord injury is on the rise globally, and unfortunately, there is no known cure for this condition [1–3].

The blood-spinal cord barrier (BSCB), similar to the blood–brain barrier, can prevent toxins, blood cells, and pathogens from entering the spinal cord and is essential for normal neurological function [4]. The occurrence of spinal cord injury can lead to the destruction of BSCB integrity, thereby disrupting the microenvironment of the spinal cord parenchyma, leading to secondary injuries such as spinal cord edema, hemorrhage, oxidative stress, and excessive inflammatory response [5, 6]. Current evidence suggests that BSCB permeability is increased after injury, leading to the exudation of macrophages from the injured blood vessels and accumulation in the spinal cord microenvironment, further increasing the permeability of BSCB. Accordingly, disruption of the spinal cord microenvironment is further aggravated [7]. In this respect, Ge et al. described the mechanism underlying the interaction between macrophages and vascular endothelial cells [8]; The infiltration of lymphocytes and neutrophils is associated with BSCB damage in SCI, lymphocyte infiltration mediates inflammation, the proliferation of reactive astrocytes, and scar formation, while neutrophils lead to demyelination and neuroinflammatory events [9, 10]. Endothelial cells, intercellular junctions, pericytes, basement membranes, and astrocytic foot processes are responsible for the barrier function of BSCB [4]. Tight junctions between endothelial cells are an essential factor affecting the permeability of BSCB, which are mainly composed of plasma membrane proteins such as occludin, claudins, zonula occludens (ZO) proteins, and junctional adhesion molecules [11, 12]. Therefore, preventing the destruction of TJs may mitigate the cascade reaction caused by BSCB dysfunction and yield a different impact on SCI repair.

Regulatory T (Treg) cells are critical regulators of inflammation, including thymic Treg cells and peripheral Treg cells, which are essential for immune tolerance and homeostasis. Regulatory T cells are CD4 + T cells with high expression of CD25, and the Foxp3 gene is the main regulator during the development of Treg cells [13]. Foxp3 + Tregs may play a role in the inflammatory microenvironment of SCI by mediating the secretion of inflammatory cytokines and promoting the anti-inflammatory phenotype of immune cells. The secretion of anti-inflammatory cytokines such as IL-10 can further enhance the balance of proinflammatory phenotype dominance in the lesion site. In addition, the reversal from a pro-to an anti-inflammatory environment improves tissue repair, reduces secondary damaged cells, and controls the inflammatory cascade and expansion [14–16]. Tregs are thought to be protective and have been reported to suppress destructive inflammatory responses in stroke models [17]. It has also been established that Treg cells have a protective role in models of immune-mediated neurodegeneration and patients with amyotrophic lateral sclerosis [18]. Our previous study also substantiated that Treg cells and their derived exosomes could promote the recovery of motor function after spinal cord injury by inhibiting microglial pyroptosis [19]. However, whether the stimulatory effect of Treg cells on SCI repair is related to BSCB has not been established, emphasizing the need for further study.

Exosomes are extracellular vesicles with an average diameter of about 100 nm, which can be taken up by remote cells, influencing their behavior and function and embodying a means of intercellular communication [20]. It has been established that communication through exosomes is involved in the pathogenesis of various diseases, including neurodegenerative and inflammatory diseases. Exosomes contain various cellular components, including DNA, RNA, lipids, metabolites, and cytoplasmic and cell surface proteins. Over the years, the role of exosomes in the pathophysiology of spinal cord injury has become a research hotspot [21]. Previous scholars have explored the role and specific mechanism of exosomes derived from human bone marrow mesenchymal stem cells, pericytes, and macrophages on BSCB [8, 22, 23]. However, whether Treg cell-derived exosomes promote BSCB repair has not been confirmed, which prompted us to conduct further investigation.

This study found significant Treg cell infiltration at the injury site on day 7 after SCI, with substantial neovascularization. However, the BSCB still maintained high permeability, and the expression of TJs-related proteins was significantly reduced, indicating that the new endothelial cells did not form a complete tight junction [24, 25]. Although the infiltration of Treg cells cannot fully reverse the complicated biological process that leads to the destruction of BSCB and result in significant improvement, we hypothesized that Treg cells might assist in promoting the restoration of TJs.

## Material and methods

### Cell culture

A mouse brain microvascular endothelial cell line (bEND.3) was purchased from Shanghai Cell Research Center (Shanghai, China). The cells were cultured in DMEM medium containing 10% PBS and 1% penicillin/streptomycin. The temperature of the cell incubator was maintained at 37 °C, and the carbon dioxide concentration was maintained at 5%.

### Exosomes isolation and identification

Exosomes were extracted from the culture medium supernatant of Treg cells. Treg cells were cultured with exosome-free medium, and the supernatants were collected by centrifugation at 300g for 10 min and at 2000 g for 10 min. Cell debris was removed by filtering the collected cell supernatant using a 0.22 μm filter at the end of centrifugation. The supernatant was further centrifuged at 4000g until the volume of the upper chamber was reduced to 200 μl. To purify exosomes, the liquid was loaded onto 30% sucrose/D2O buffer and ultracentrifuged at 100,000g for 60 min at 4 °C using an impound ultracentrifuge. To identify the extracted exosomes, the morphology, diameter distribution, and number of exosomes were observed using transmission electron microscopy (TEM) and nanoparticle tracking analysis (NTA). Western blotting was used to detect exosome surface markers (CD9, CD63, CD81).

### Measurement of transepithelial electrical resistance (TEER) and permeability detection

Measurements of TEER and FITC-dextran permeability evaluation were performed as previously described [26].

bEnd.3 cells were cultured in vitro by Transwell system. TEER values of OGD/R, T-CM and control groups were measured by Millcell ERS-2 (Merk-Millipore). To accurately calculate TEER (Ω × cm^2^), the resistance obtained by inserting the membrane (blank resistance) was subtracted from the reading obtained by inserting the cell (sample resistance). This value is multiplied by the surface area of the insert (0.33 cm^2^).

bEnd.3 cells were treated with OGD/R or T-CM, 1 mg/mL FITC-dextran (serum-free DMEM) was added to the inserts of the Transwell system and incubated at 37 °C for 30 min. After that, 100 μL of the sample were transferred into 96-well plates with black walls, and the amount of FITC-dextran accumulated was assessed using Envision fluorescence microplates.

### Animals

The experimental mice were female, clean-grade C57BL/6 J mice, weighing 20–25 g and aged 8–10 weeks. Foxp3^DTR^ (Foxp3 gene locus knock-in diphtheria toxin receptor) mice that can use DT knockout Treg cells were provided by Shanghai Nanfang Model Biotechnology Co., LTD (Shanghai, China). All animal experiments were approved by the Animal Committee of the Affiliated Jiangsu Shengze Hospital of Nanjing Medical University.

### Treg cell depletion and adoptive transfer

Treg cells were depleted in Foxp3^DTR^ mice by intraperitoneal injection of diphtheria toxin (DT, ip, 0.05 μg/g body weight) every three days from 3 days before SCI to 28 days after SCI. Spleens, inguinal, and axillary lymph nodes of healthy mice were collected to prepare a single-cell suspension. A Treg cell isolation kit (Miltenyi Biotec) was used to isolate CD4 + CD25 + Treg cells, and CD4 + cells were negative selected and CD25 + cells were positive selected. To transfer Treg cells into vivo, transfer was accomplished by injecting 3 × 10^6^ Treg cells into SCI mice via the tail vein of the mice. Control mice received an equal volume of PBS.

### Permeability assays in vivo

Injection of FITC-dextran: SCI mice were selected and fixed, and their tails were soaked in warm saline for 20 min to dilate the tail vein. The tail of the mice was wiped with alcohol, and the tail vein was punctured along the middle and posterior thirds of the dorsal tail vein of the mice with an insulin syringe (13G). After a successful puncture, 200 μl FITC-dextran were slowly injected. Once the injection was completed, the injection point was pressed for 1 min and sterilized. After the end of the injection, the mice were returned to the cage, and the spinal cord was harvested 1 h later. Spinal cord tissues injected with FITC-dextran should be appropriately protected from light to avoid FITC-dextran quenching during sampling and sectioning. CD31 was used to mark the blood vessels, and the leakage of FITC-dextran was observed. Fluorescence intensity was quantified using ImageJ software.

### Footprint analysis

Analyses of footprints were performed on day 28 post-injury mice to assess motor function recovery. Blue dye was used to mark the front paw and red dye was used to mark the back paw, and mice were prompted to run in a straight line on the prepared test paper. To assess the weight support and gait of the mice, a 4-point scoring system was used as described previously [27].

### Swimming test

Swimming tests were performed on mice before and after SCI, in which mice were placed in a tank and encouraged to swim from one end of the tank to the other. swimming postures were recorded, and motor function recovery was assessed using the Louisville Swim Scale.

### Basso mouse scale (BMS) behavioral analysis

The range of motion of hind limbs, trunk position and stability, coordination of front and back limbs, paw position, toe space, and tail position were measured before spinal cord injury and 1, 3, 7, 14, 21, and 28 days after injury. The BMS score was used to evaluate motor function. Ensure that the recorder was unaware of the group assignments during the observation period.

### Electrophysiology testing

Electrophysiological tests were performed as described previously [19], with stimulating electrodes placed at the anterior end of the surgically exposed spinal cord, recording electrodes placed at the flexor muscle of the biceps femoris, reference electrodes placed at the distal tendon of the hind limb muscles, and ground electrodes placed subcutaneously. Stimulation with a single square wave (10 mA, 0.5 ms1 Hz).

### Western blot assay

Target cells or tissues were prepared, and proteins were extracted after lysis using RIPA lysate. Protein concentrations were analyzed by BCA assay. Equal amounts of proteins were separated using SDS-PAGE gels and transferred to PVDF membranes. Upon completion of the transfer, the membranes were blocked with BSA blocking solution (room temperature, 1 h), followed by incubation with primary antibodies overnight at 4 °C. The next day, the PVDF membrane was washed with TBST and incubated with secondary antibodies (room temperature, 2 h), and finally the target protein bands were visualized using the ECL reagent.

### RNA extraction and qRT-PCR

Total RNA from cells and exosomes was extracted using TRIzol reagent (Invitrogen, Carlsbad, CA, USA), the hairpin itTM miRNA qPCR quantification kit (GenePharma, China), and PrimeScript RT kit (Takara, Japan) to synthesize cDNA for miRNA and mRNA. Finally, qRT-PCR analysis was performed with the TB Green^®^ Premix Ex TaqTM kit (Takara, Japan). Expression levels were normalized against an internal control (GAPDH or U6), and relative expression levels were assessed using the 2 -ΔΔCT method.

### Immunofluorescence staining

Mouse spinal cord tissues were obtained after cardiac perfusion, fixed with 4% paraformaldehyde, sectioned, blocked with 10% BSA, incubated with primary antibodies (Claudin-5, Occludin, ZO-1, and CD31) at 4 ℃ overnight, and incubated with corresponding secondary antibodies at room temperature for 1 h the next day. The nuclei were labeled with the DAPI reagent. The cells were fixed with 4% paraformaldehyde and permeabilized with 0.05% Triton X-100. After blocking, the cells were incubated with the primary antibody (Occludin, ZO-1) at 4 ℃ overnight, and treated with the corresponding secondary antibody and DAPI the next day. The staining was observed using a fluorescence microscope.

### Luciferase reporter assay

The target genes of miR-2861 were selected through the TargetScan (www.targetscan.org) database. IRAK1-3 'untranslated region (UTR) was developed by Hippo Biotechnology Co., LTD. constructed, and containing wild-type (WT) or mutated (MUT) miR-2861 binding sequences (Nanjing, China). These sequences were cloned into the FseI and XbaI restriction sites of the pGL3 luciferase control reporter vector (Promega, USA) in order to construct IRAK1 3'-UTR reporter vectors (pGL3-WT-IRAK1 and pGL3-mut-IRAK1). bEND.3 cells were seeded in 24-well plates, and transfection was initiated after 24 h of incubation. Finally, transfected bEND.3 cells or negative control cells were seeded in 96-well plates, and 100 ng of pGL3-WT-IRAK1 or pGL3-MUT-IRAK1 3'-UTR was also added to 96-well plates. The results were analyzed by a dual luciferase reporter assay system.

## Statistical analyses

Data were expressed as mean ± standard deviation (mean ± SD) and derived from more than three repeated experiments. GraphPad Prism 8.0 software was used for statistical analysis. Significant differences were determined using the Student's t-test or one-way or two-way ANOVA. The difference was statistically significant at p value < 0.05. (*p < 0.05; **p < 0.01; ***p < 0.001).

## Result

### Damage to the blood-spinal barrier integrity and Treg cell infiltration at the injury site after spinal cord injury

**Fig. 1:**
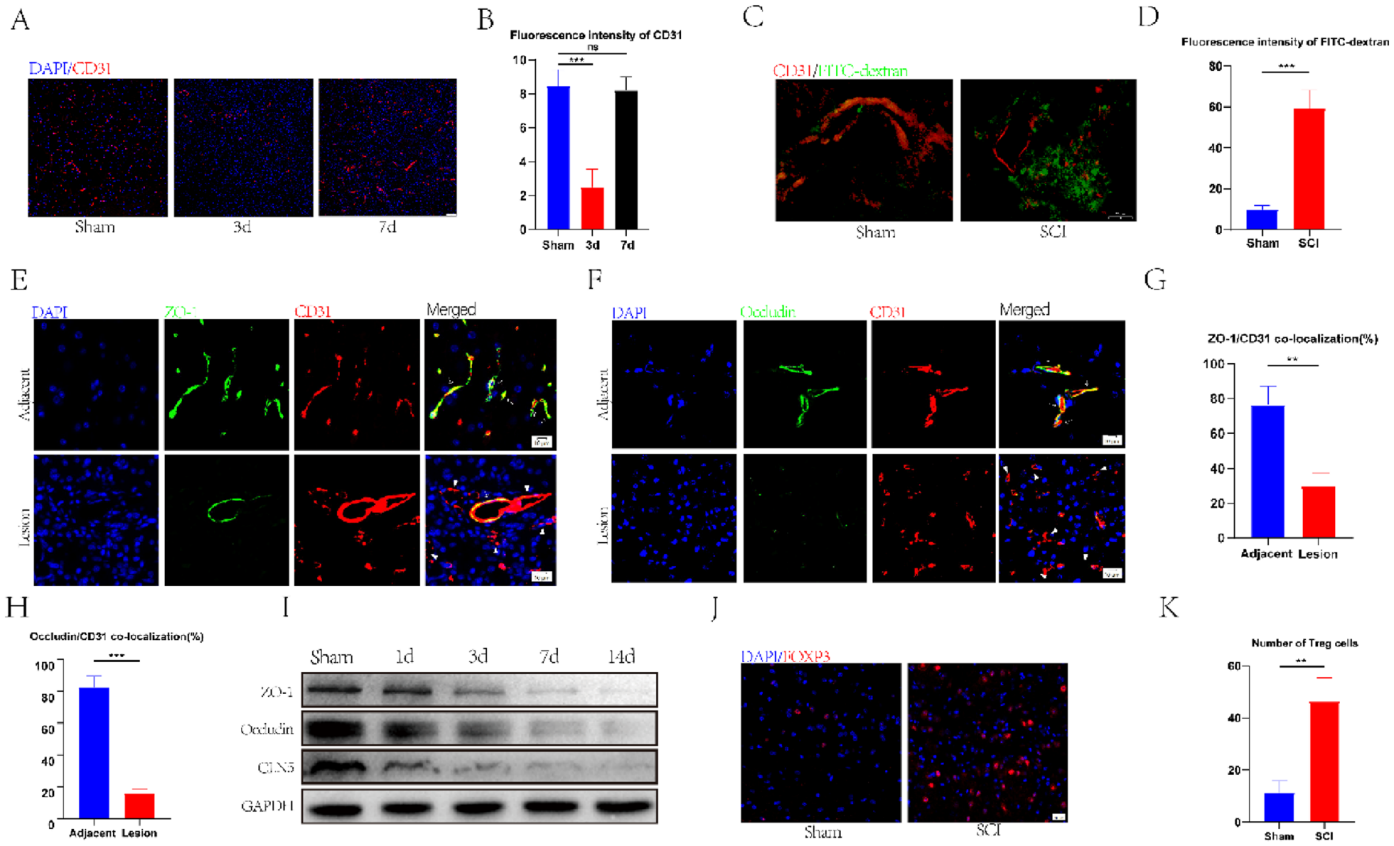
BSCBS and TJs were destroyed, and Treg cell infiltration increased after spinal cord injury. **A**, **B** The distribution and quantification of blood vessels labeled with CD31 (n = 3). **C, D** Fluorescence intensity and quantification of FITC-dextran around CD31-labeled blood vessels on day 7 after spinal cord injury (n = 4). **E–H** co-immunostaining and quantification of TJS-related proteins and vascular at injury sites and adjacent sites on day 7 after spinal cord injury (arrows: intact blood vessels; triangles: damaged blood vessels) (n = 3). **I** Western blotting detected the expression of TJs protein at each time point after spinal cord injury. **J, K** Representative staining and quantification of Foxp3-labeled Treg cells at the injured site after spinal cord injury (n = 3)

In this study, we utilized CD31 labeling to track the blood vessels in both healthy spinal cord tissue and the site of injury to monitor vascular recovery and any disruption to the blood-spinal cord barrier following spinal cord injury (Fig. 1A). We found that the vascular density at the injury site was lower than in the normal spinal cord tissue on day 3 (Fig. 1A, B). There was no significant difference in the density of blood vessels between the injury site and the normal spinal cord tissue on day 7 (Fig. 1A, B). These results substantiated that the blood vessels at the injury site began to repair on day 3 after spinal cord injury and reached a steady state on day 7. Next, FITC-dextran was injected into mice with spinal cord injury and normal mice, and its fluorescence intensity was quantified to observe the status of the blood-spinal barrier after spinal cord injury. We found that the fluorescence intensity at the site of injury in the SCI group was significantly increased compared to the normal group (Fig. 1C, D), implying significant damage to the BSCB, leading to the penetration of FITC-dextran following SCI. Immunofluorescence staining was performed on the proteins associated with the tight junction of blood vessels in the injury and non-injury areas of spinal cord tissue after SCI to observe the tight junctions of blood vessels. Our findings indicated a significant decrease in the colocalization of ZO-1 and Occludin with CD31 in the injury area compared to the non-injury areas (Fig. 1E–H). Meanwhile, western blotting showed that the expression of TJS-related proteins gradually decreased (Fig. 1F). These results substantiated that the integrity of BSCB in the injury area after SCI was affected and gradually exacerbated. Immunofluorescence experiments revealed significant infiltration of Treg cells at the injury site after SCI (Fig. 1H, I). Accordingly, we speculated that Treg cells could influence the repair of BSCB.

### Treg cells promote BSCB repair and motor function recovery in SCI mice

**Fig. 2:**
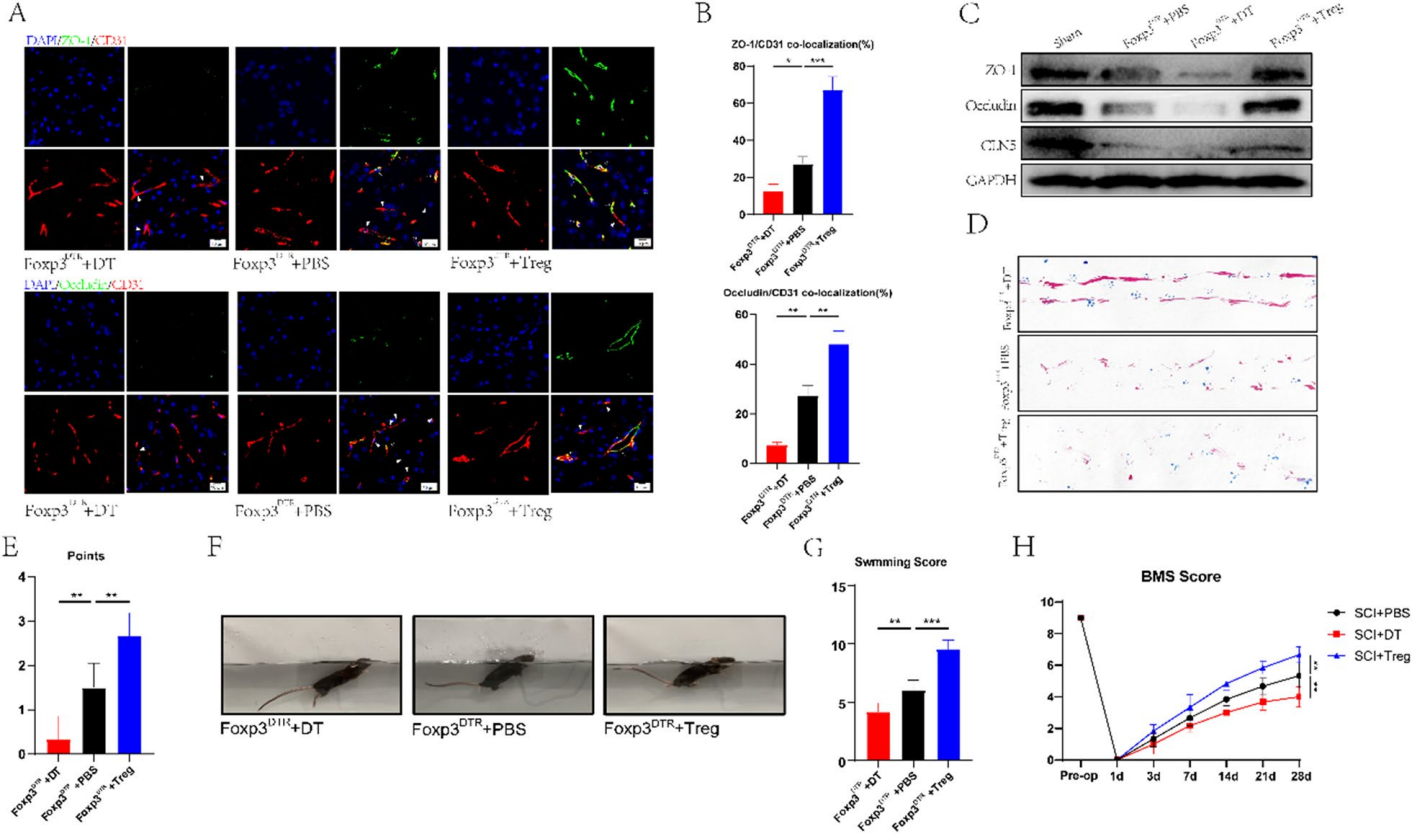
The infiltration of Treg cells in the spinal cord can help the recovery of TJs protein expression and motor function in mice with spinal cord injury. **A, B** Co-immunostaining and quantification of TJs protein and blood vessels at the injured site on day 7 after spinal cord injury (arrows: intact blood vessels; triangles: damaged blood vessels) (n = 3). **C** The expression of TJs protein in the spinal cord of the sham group, PBS group, Treg cell knockout group, and transplantation group was detected by western blotting. **D, E** Footprint analysis showed the recovery of motor function at 28 days after spinal cord injury in Treg cells knocked out and transplanted mice (n = 6). **F** Swimming tests showed recovery of motor function 28 days after spinal cord injury in Treg cells knocked out and transplanted mice. **G** Louisville Swim Scale in Treg cells knocked out and transplanted in mice (n = 6). **H** BMS scores showed the recovery of motor function at 28 days after spinal cord injury in Treg cells knocked out and transplanted in mice (n = 6)

To explore the effect of Treg cells on BSCB repair, we first established Foxp3^DTR^ transgenic mice and selectively knocked out Treg cells by injecting diphtheria toxin (DT) and verified the knockout efficiency (Additional file 1: Fig. S1A, B). After injecting Treg cells into the tail vein of Foxp3^DTR^ transgenic mice, Treg cell overexpression mice models were established. We stained the spinal cord TJs and blood vessels of SCI mice in the Foxp3^DTR^ + DT group, Foxp3^DTR^ + PBS group, and Foxp3^DTR^ + Treg group by Occludin, ZO-1, and CD31, respectively. The colocalization of TJs protein and blood vessels in the Foxp3DTR + DT group was lower than in Foxp3^DTR^ + PBS and Foxp3^DTR^ + Treg groups (Fig. 2A, B), while significantly higher levels were observed in the Foxp3^DTR^ + Treg group than the other two groups (Fig. 2A, B). These results indicate that the knockout of Treg cells could inhibit the expression of TJs-related proteins in SCI mice spinal cord, and the overexpression of Treg cells can help in better colocalization of TJs proteins and blood vessels, consistent with the Western blotting results (Fig. 2C). To explore the effects of different treatment methods on motor function of SCI mice, footprint analysis was conducted on three groups of SCI mice respectively. The Foxp3^DTR^ + DT group experienced the worst footprint analysis results, followed by the Foxp3^DTR^ + PBS group, and the Foxp3^DTR^ + Treg group (Fig. 2D, E). The Swimming test and Basso mouse scale (BMS) behavioral analysis yielded similar outcomes (Fig. 2F–H). These results confirmed the effect of Treg cells on the motor function of SCI mice. According to the above results, we advocate that the accumulation of Treg cells at the injury site can promote the recovery of BSCB in mice after SCI and improve motor function.

### Treg-derived exosomes promoted the expression of tight junction protein in bEND.3 cells

**Fig. 3:**
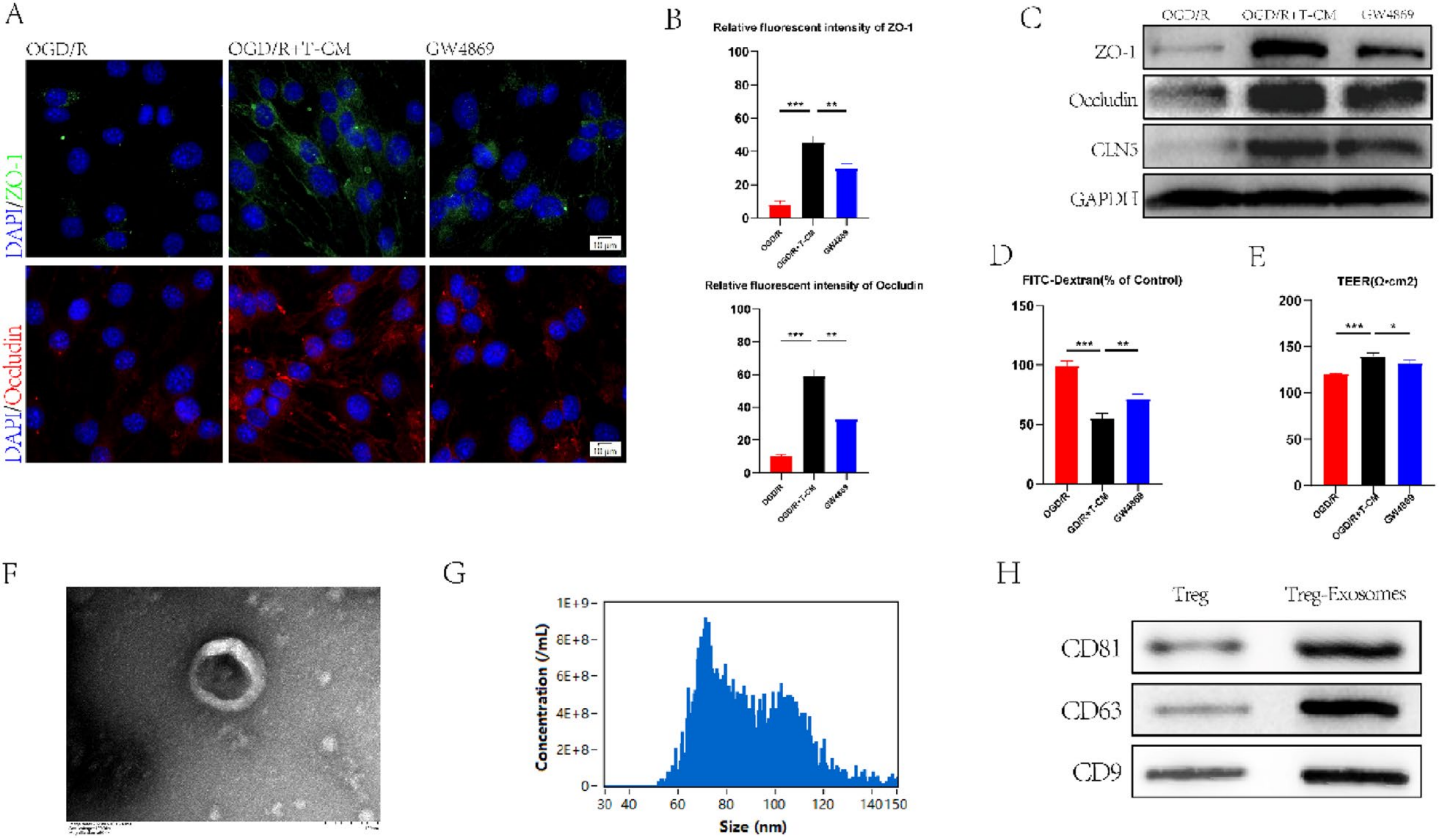
Treg-Exos promote the expression of TJs protein in vitro. **A, B** Immunofluorescence staining and quantization of TJs protein in the OGD/R group, the OGD/R + T-CM group, and the GW4869 group (n = 3). **C** The expression of TJs protein in three groups of bEND.3 cells were detected by western blotting. **D** TJs integrity of bEND.3 cells in each group were evaluated by FITC-Dextran (n = 3). **E** TJs integrity of bEND.3 cells in each group were evaluated by TEER (n = 3). **F**–**H** TEM, NTA analysis, and Western blot were used to identify exosomes

To further explore the mechanism of Treg cells on BSCB in SCI mice, the BSCB damage model was constructed in vitro by oxygen–glucose deprivation/reoxygenation (OGD/R) treatment of bEnd.3 cells to simulate BSCB damage in vivo, and immunofluorescence staining was performed. Conditional medium obtained from Treg cells(T-CM) pretreated with anti-CD3 (1ug/ml) and anti-CD28 (10ug/ml) were collected and co-cultured with pretreated bEND.3. The experimental results showed that compared with bEnd.3 cells treated with OGD/R alone, the expression of Occludin and ZO-1 protein in the T-CM treatment group was significantly increased (Fig. 3A, B). The exosomes secreted by cells have been documented to play a role in cell communication. To further explore the effect of T-CM, bEND.3 cells co-cultured with T-CM were treated with an exosome inhibitor GW4869. The addition of GW4869 reversed the stimulatory effect of T-CM on the expression of TJs protein (Fig. 3A, B). The above results were verified by western blotting (Fig. 3C). Endothelial permeability and transendothelial electrical resistance (TEER) were used to assess the therapeutic effects of exosomes (Fig. 3D, E). The above evidence confirmed the conjecture that Treg cells could affect the expression of TJs protein in bEND.3 cells by secreting exosomes. In previous experiments [19], we identified Treg cell-derived exosomes by transmission electron microscopy (TEM), and nanoparticle tracking analysis (NTA) (Fig. 3F, G) and verified them by western blotting (Fig. 3H). Exosomes labeled using Dil can be ingested by bEND.3 cells (Additional file 1: Fig. S2.A).

### Treg-derived exosomes promote BSCB repair and motor function recovery in SCI mice

**Fig. 4:**
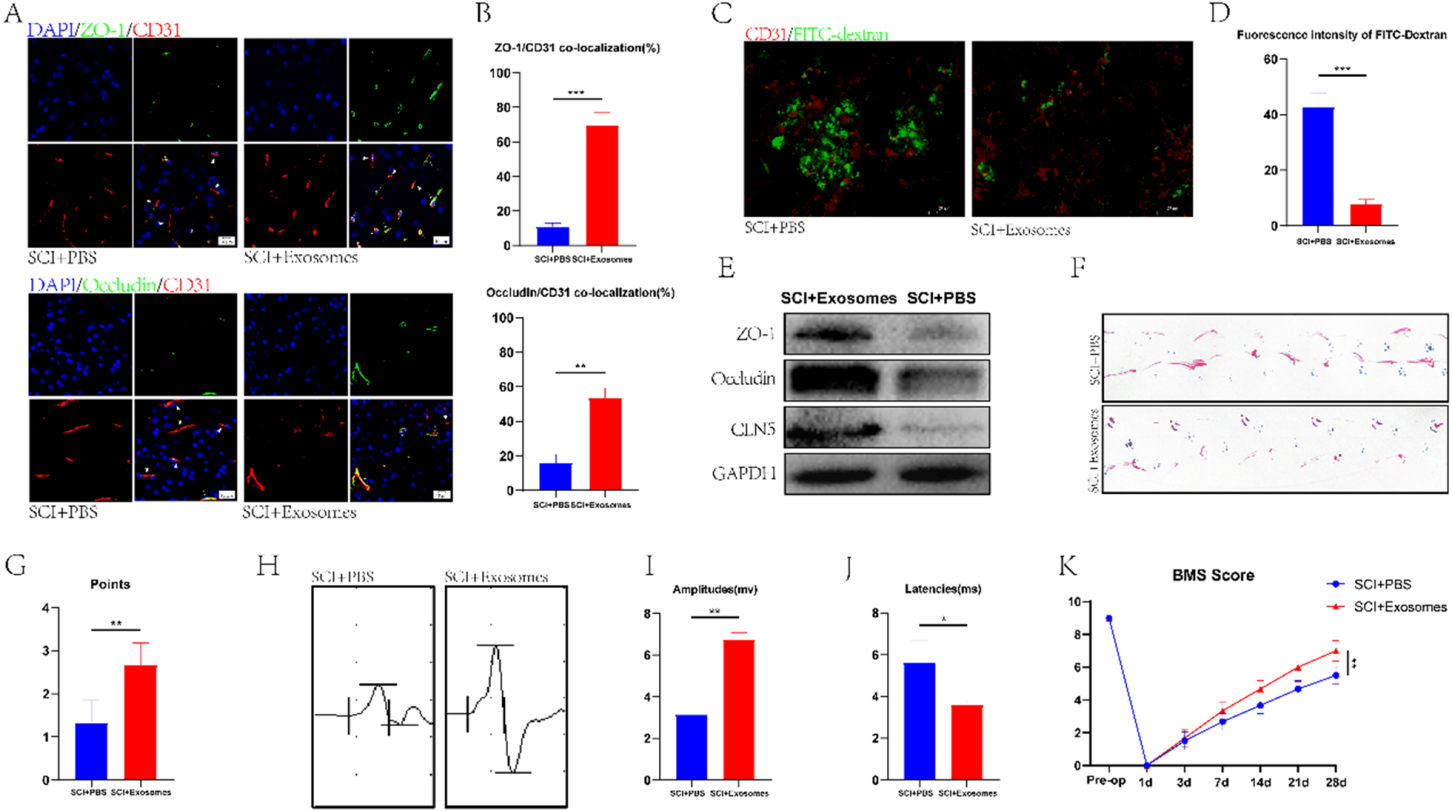
Treg-Exos promote the expression of TJs protein in vivo and improve the exercise ability of mice. **A, B** Co-immunostaining and quantification of TJs protein and blood vessels in the spinal cord of mice on day 7 after spinal cord injury (arrows: intact blood vessels; triangles: damaged blood vessels) (n = 3). **C, D** The fluorescence intensity and quantification of FITC-dextran in the spinal cord at day 7 after spinal cord injury were used to detect the permeability of BSCB (n = 4). **E** Western blot was used to detect the expression of TJs protein in the spinal cord after Treg-Exos intervention. **F–K** Footprint analysis (n = 6), MEP analysis (n = 3), and BMS scores (n = 6) were used to evaluate the locomotor ability at day 28 after Treg-Exos intervention in spinal cord injury mice

To explore whether Treg-derived exosomes (Treg-Exos) can play a role in BSCB repair in SCI mice, isolated exosomes were injected into SCI mice, followed by immunofluorescence staining on the spinal cord of the mice. The colocalization of Occludin and ZO-1 with CD31, was more obvious in the SCI + exosomes group compared to the SCI + PBS group. (Fig. 4A, B). These results indicated that BSCB repair was more significant in SCI mice after exosome injection, similar to the findings of the FITC-dextran penetration experiment and western blot (Fig. 4C–E). We compared the motor function of the SCI + exosomes group with the SCI + PBS group by multiple behavioral analyses and found that the motor function of the former group was significantly improved after exosomal treatment compared to the latter (Fig. 4F–K).

### Exosomes contain miR-2861 and can deliver it to bEnd.3 cells

Given that exosomes can deliver miRNAs and play regulatory roles, we selected the TOP20 miRNAs from Treg cells and exosomes derived from Treg from GEO database, and obtained 5 miRNAs after intersection, among which miR-2861 was selected for further study (Additional file 1: Fig. S3A). The presence of miR-2861 in exosome-treated bEnd.3 cells were verified by qRT-PCR (Additional file 1: Fig. S3B).

### Exosomes deliver miR-2861 in vitro to act on bEND.3 cells to improve TJs protein expression

**Fig. 5:**
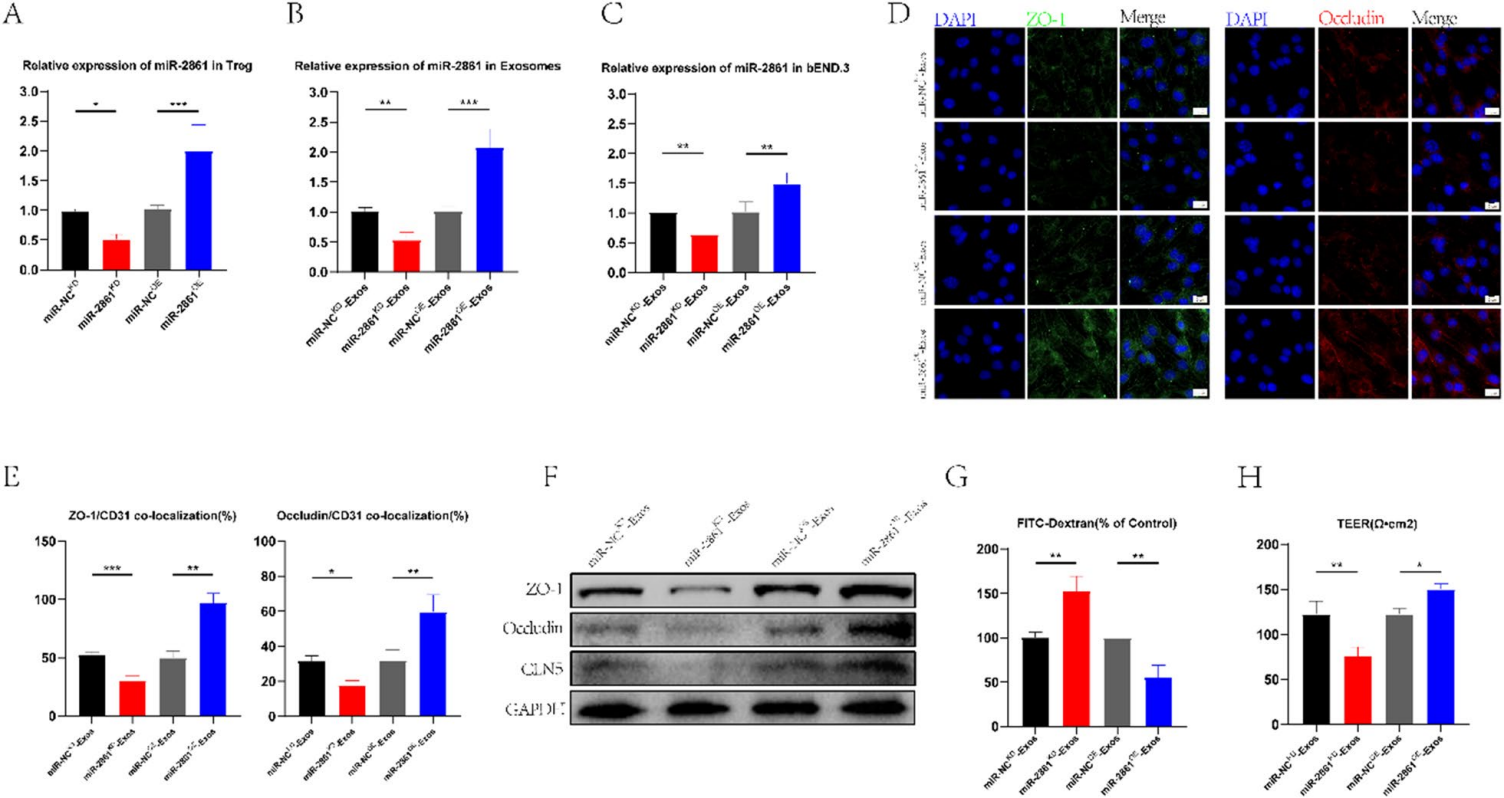
Treg-Exos promote TJs protein expression in vitro by delivering miR-2861. **A** The transfection efficiency of miR-2861 in Treg cells was verified by qRT-PCR. **B** The relative expression of miR-2861 in Treg-Exos. **C** The relative expression of miR-2861 in miR-NC^KD^-Exos, miR-2861^KD^-Exos, miR-NC^OE^-Exos, and miR-2861^OE^-Exos-treated bEND.3 cells. **D, E** Expression and quantification of TJs protein in miR-NC^KD^-Exos, miR-2861^KD^-Exos, miR-NC^OE^-Exos, and miR-2861^OE^-Exos-treated bEND.3 cells. **F** Western blotting was used to detect the expression of TJs protein in miR-NC^KD^-Exos, miR-2861^KD^-Exos, miR-NC^OE^-Exos, and miR-2861^OE^-Exos-treated bEND.3 cells. **G, H** FITC-dextran and TEER values were used to assess the TJs permeability of miR-NC^KD^-Exos, miR-2861^KD^-Exos, miR-NC^OE^-Exos, and miR-2861^OE^-Exos-treated bEND.3 cells (n = 3)

After establishing the existence of miR-2861 in exosomes, we sought to confirm whether exosome-laden miR-2861 could play a critical biological role in BSCB recovery. Therefore, we knocked down and overexpressed miR-2861 by lentivirus transfection and constructed corresponding negative controls (miR-2861^KD^, miR-NC^KD^, miR-2861^OE^, and miR-NC^OE^), respectively. Transfection efficiency was verified by qRT-PCR (Fig. 5A). To observe the effects of miR-2861 at the cellular level, we extracted exosomes (miR-NC^KD^-Exos, miR-2861^KD^-Exos, miR-NC^OE^-Exos, and miR-2861^OE^-Exos) from lentivirus-transfected Treg cell models, and exosomes were co-cultured with OGD/ R-treated bEnd.3 cells. We found that the expression of miR-2861 in the miR-2861^KD^-Exos group was significantly lower than that in the miR-NC^KD^-Exos group. Moreover, miR-2861 expression in the miR-2861^OE^-Exos group was significantly higher than in the miR-NC^OE^-Exos group(Fig. 5B). In bEnd.3 cells, the expression of miR-2861 in each group exhibited the same trend, which corresponded to the expression level of miR-2861 in the exosome group (Fig. 5C). Immunofluorescence staining showed that the fluorescence intensity of Occludin and ZO-1 in the miR-2861^KD^-Exos group was significantly lower than in the miR-NC^KD^-Exos group. The opposite results were observed in the miR-2861^OE^-Exos group (Fig. 5D, E), consistent with WB (Fig. 5F). These results suggested the knockdown and overexpression of miR2861 could potentially reduce and increase the expression of TJs protein. We also observed the permeability and TEER of bEND.3 cells and found that the permeability was increased in the miR-2861^KD^-Exos group and decreased in the miR-2861^OE^-Exos group (Fig. 5G). TEER was significantly decreased in the miR-2861^KD^-Exos group and increased in miR-2861^OE^-Exos group (Fig. 5H). The above results provided compelling evidence that miR-2861 is a biological mediator that can promote BSCB repair, and it is of great significance to explore the underlying mechanisms.

### miR-2861 can repair the BSCB and increase motor function in vivo

**Fig. 6:**
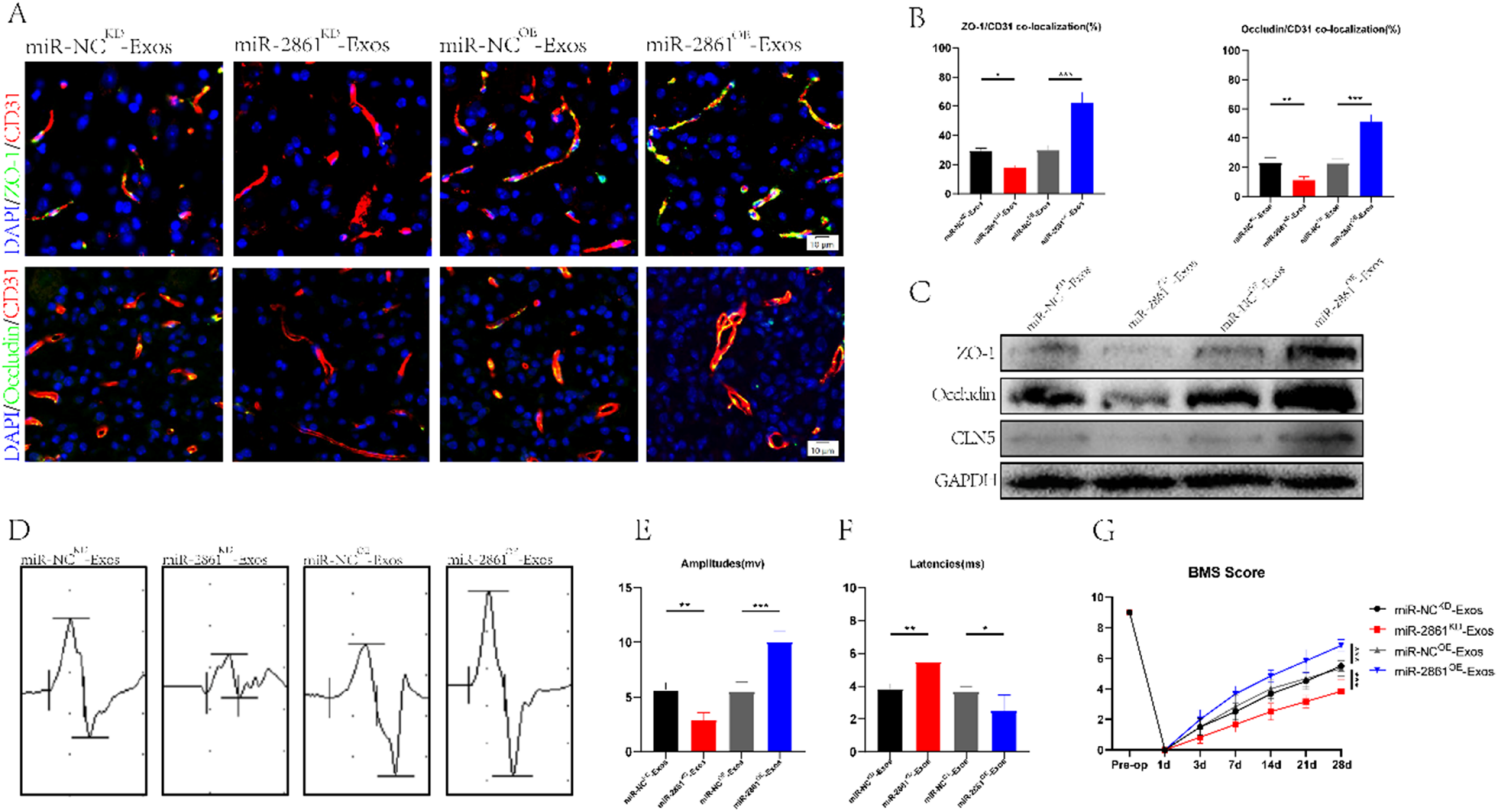
Treg-Exos promote BSCB repair in vivo by delivering miR-2861. **A, B** Co-immunostaining and quantification of TJs protein and blood vessels at the injured site in the spinal cord on day 7 after spinal cord injury (n = 3). **C** Western blotting was used to detect the expression of the TJs protein of the spinal cord in miR-NC^KD^-Exos, miR-2861^KD^-Exos, miR-NC^OE^-Exos, and miR-2861^OE^-Exos-treated mice. **D–G** MEP analysis (n = 3) and BMS score (n = 6) were used to evaluate the recovery of motor ability at day 28 after the intervention of miR-NC^KD^-Exos, miR-2861^KD^-Exos, miR-NC^OE^-Exos, and miR-2861^OE^-Exos in mice with spinal cord injury

After validating the role of miR-2861 at the cellular level, we sought to clarify the function of miR-2861 in SCI mice in vivo. We grouped exosomes into miR-2861^KD^-Exos, miR-NC^KD^-Exos, miR-2861^OE^-Exos, and miR-NC^OE^-Exos and injected them into SCI mice. We found that the colocalization intensity of TJs protein and blood vessels in the miR-2861^KD^-Exos group was significantly lower than in the miR-NC^KD^-Exos group, while the colocalization intensity of TJS protein and blood vessels in miR-2861^OE^-Exos group was higher than in the miR-NC^OE^-Exos group (Fig. 6A, B). This finding was supported by Western blot results (Fig. 6C). Behavioral analysis experiments were used to observe the improvement of motor function in SCI mice. MEP and BMS score analyses showed that low expression of miR-2861 was detrimental to the recovery of motor function, while high expression of miR-2861 could enhance motor function (Fig. 6D–G). Overall, we confirmed that miR-2861 could be transported by exosomes and regulate the repair of BSCB, thereby improving motor function.

### miR-2861 can negatively regulate IRAK1

**Fig. 7:**
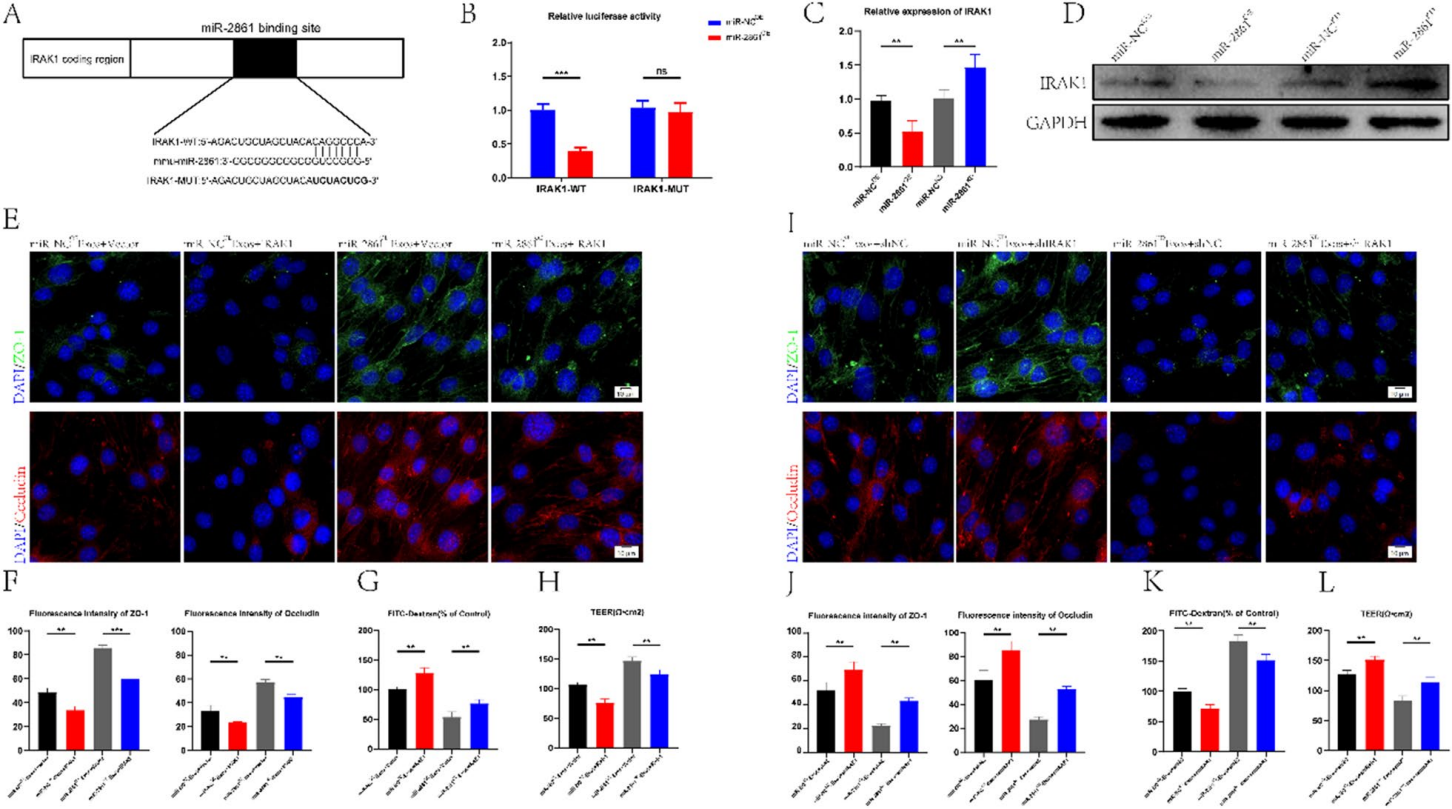
miR-2861 promotes BSCB repair by inhibiting IRAK1 expression. **A** miR-2861 in Treg-Exos can bind to the 3'-UTR to inhibit IRAK1 expression. **B** A luciferase report assay was used to verify IRAK1 as a downstream target gene of miR-2861 (n = 3). **C** qRT-PCR and western blot were used to verify that miR-2861 could negatively regulate the relative expression of IRAK1 (n = 3). **E**, **F** Expression and quantification of TJs protein in Treg-Exos-treated bEND.3 cells. **G**, **H** FITC-dextran and TEER values were used to assess the TJs permeability of Treg-Exos-treated bEND.3 cells (n = 3). **I**, **J** Expression and quantification of TJs protein in Treg-Exos-treated bEND.3 cells. **K, L** FITC-dextran and TEER values were used to assess the TJs permeability of Treg-Exos-treated bEND.3 cells (n = 3)

To further explore the specific mechanism of miR-2861, we analyzed and predicted the potential target genes of miR-2861 using an online database. Among these, IRAK1 has been associated with inflammation, and its 3' untranslated region (3' UTR) may be recognized and bound by miR-2861. To validate our conjecture, IRAK1 wild-type (WT) and mutant (MUT) 3 '-UTR sequences were constructed based on IRAK1 potential binding sites (Fig. 7A) and co-transfected with miR-2861 sequences into bEnd.3 cells. Luciferase reporter analysis revealed that when the WT-IRAK1-3'UTR and miR-2861^OE^ group were co-transfected, the luciferase activity was significantly lower than in the WT-IRAK1-3'UTR and miR-NC^OE^ group. However, when MUT-IRAK1-3'UTR and miR-2861^OE^ were co-transfected, the luciferase activity was not significantly different from the control group (Fig. 7B), indicating that the 3'UTR of IRAK1 could specifically bind to miR-2861. We further verified that IRAK1 could be negatively regulated by miR-2861 by qRT-PCR and Western blot (Fig. 7C, D).

### miR-2681 affects BSCB integrity by targeting IRAK1

Loss and gain-of-function experiments were designed to further investigate the relationship between miR-2861 and IRAK1 and the effect of IRAK1 on BSCB. bEnd.3 cells overexpressing IRAK1 were treated with miR-NC^OE^-Exos and miR-2861^OE^-Exos, and after knocking down IRAK1, bEnd.3 cells were treated with miR-NC^KD^-Exos and miR-2861^KD^-Exos. The results showed that IRAK1 overexpression reversed the high expression of TJs protein induced by miR-NC^OE^-Exos and miR-2861^OE^-Exos (Fig. 7E, F). IRAK1 overexpression also reversed the exosome-induced decrease in FITC-dextran permeability and increase in TEER (Fig. 7G, H). Similarly, we found that knocking down IRAK1 abolished the deleterious effect of miR-NC^KD^-Exos or miR-2861^KD^-Exos on TJs (Fig. 7I, J). FITC-dextran permeability and TEER values yielded similar results (Fig. 7K, L). Collectively, the above data suggest that miR-2681 can target IRAK1 to affect BSCB integrity.

## Discussion

Damage to the vasculature and disruption of BSCB integrity are common consequences of SCI [28]. It has been reported that after spinal cord injury, the capillaries undergo mechanical rupture, and molecules and cells carried by the blood quickly enter the damaged parenchyma. The pathological cascade further aggravates the spinal cord injury and leads to a secondary inflammatory response, affecting the remodeling of TJs of neovascularization and resulting in persistent hyperpermeability of BSCB. Thus, the impaired state of BSCB after SCI persists for up to 56 days, although BSCB dysfunction occurs within 5 min of SCI [29, 30]. This study confirmed substantial neovascularization at the injury site on day 7 after SCI; however, BSCB maintained high permeability, and the expression of ZO-1, Occludin, and CLN-5 decreased significantly with time; this suggests that nascent endothelial cells do not form complete tight junctions [24, 25, 31]. In addition, we found that the expression of Occludin and ZO-1 in the injured spinal cord was significantly lower than that in the normal spinal cord after spinal cord injury. Consistent results were obtained by observing endothelial vascular permeability with FITC-Dextran. These phenomena suggest that neovascularization with incomplete TJs in the injury area contributes to the high permeability of BSCB. Interestingly, we found a significant accumulation of Treg cells at the injury site on day 7 after SCI in mice. Based on our previous study that Treg cells could promote the recovery of SCI, we hypothesized that the infiltration of Treg cells into the injury area could restore the barrier function of BSCB. Indeed, exploring the mechanism of Treg cells in regulating BSCB after spinal cord injury can provide an important reference for treating this patient population in the future.

The protective role of Treg cells in many diseases, especially the central nervous system, has been established. Some scholars have found that Treg cell-derived osteopontin could promote microglia-mediated white matter repair after ischemic stroke, and increasing the abundance of endogenous Treg cells yielded a protective effect on the brain 1–7 days after ischemic injury [32]. It has been demonstrated that Treg cells could mediate the recovery of EAE by controlling the proliferation and motility of effector T cells in the central nervous system. Moreover, regulatory T cells could promote remyelination in the central nervous system and regulate neuroinflammation and microglial pyroptosis. Treg cells can also secrete a potent anti-inflammatory cytokine: IL-10, mediating Treg-induced blood flow restoration and neovascularization improvement after ischemia [33–37]. Based on the above findings and the spatiotemporal relationship between Treg cells and BSCB damage, we suggest that Treg cells may affect the integrity of BSCB. After knocking out Treg cells in mice, more severe BSCB infiltration was observed compared with the control group, while the infiltration of BSCB was improved after increasing the abundance of Treg cells in mice. Similarly, the motor function of mice was also altered. The knockout of Treg cells caused exacerbation of the motor function of mice, while the increase of Treg cells yielded the opposite effect. These results support our hypothesis that Treg cells contribute to improving BSCB integrity.

Exosomes play a vital role in cell interaction and have been extensively researched for their involvement in angiogenesis following SCI. For example, human urine stem cell-derived exosomes can deliver ANGPTL3 protein to the SCI region via BSCBS to stimulate angiogenesis, and vascular endothelial cells can enhance the uptake of exosomes from hypoxia-treated MSCs, thereby activating the protein kinase A (PKA) signaling pathway to promote VEGF expression to promote angiogenesis. During SCI recovery, pericytes can be modulated by BMSCS-derived exosomes to enhance BSCB integrity [38]. Exosomes secreted by Treg cells can regulate homeostasis, inhibit microglial pyroptosis, and reduce spinal cord inflammation [39]. Therefore, we suggest that these exosomes play a critical role in regulating BSCB integrity by Treg cells. Herein, we established a spinal cord injury cell model by OGD/R treatment of bEnd.3 cells. After co-culture with the supernatant containing exosomes, the expression of Occludin and ZO-1 proteins in the cells was significantly increased, and this phenomenon was reversed after treatment with an exosome inhibitor. This result indicates that Treg cell-derived exosomes can indeed regulate the expression of TJs in bEnd.3 cells. During our bioinformatics analysis, MiR-2861 was detected in exosomes derived from Treg cells. There is a vast literature available substantiating that MiR-2861 plays a role in promoting angiogenesis, resulting in improved bone regeneration in areas of bone defects [40, 41]. Our study addresses the lack of understanding surrounding the regulation of endothelial barrier function following angiogenesis. We overexpressed and knocked down miR-2861 in Treg cells, respectively, and found the same expression trend in Treg exosomes and bEnd.3 cells treated with exosomes. Subsequently, immunofluorescence staining showed that bEnd.3 cells with high miR-2861 expression produced more Occludin and ZO-1 proteins than the control group, while bEnd.3 cells produced significantly lower Occludin and ZO-1 proteins after the knockdown of miR-2861.

Interleukin-1 receptor-activated kinase 1 (IRAK1), a serine-threonine kinase belonging to the interleukin-1 receptor-activated kinases family, is a key mediator in the TLRs/ IL-1Rs signaling pathway. It can play a vital role in the pathophysiology of cancer and inflammatory diseases [42–44]. It has been confirmed that inhibition of IRAK1 can reduce inflammation in endothelial cells. During spinal cord injury, inhibition of IRAK1 expression can inhibit the secretion of proinflammatory cytokines in spinal cord injury models [45, 46]. Interestingly, we experimentally confirmed that IRAK1 was a downstream target gene of miR-2861 and was negatively regulated by miR-2861. Then the rescue experiments showed that miR-2861 could enhance BSCB integrity by inhibiting IRAK1 expression, which explains how Treg cells regulate BSCB recovery through exosomes.

## Conclusion

Our study revealed Treg cell infiltration of the injury site after SCI. The exosomes secreted by Treg cells could promote the repair of BSCB and reduce their permeability, thereby reducing the excitation injury caused by infiltration and promoting the recovery of motor function. Overall, our findings reveal a new target for treating SCI. Albeit the involvement of Treg cells in BSCB restoration was substantiated, it is highly conceivable that other cells or molecules participate in this process, warranting further research.

### Supplementary Information

**Additional file 1: Fig. S1.** Validation of the knockout efficiency of Treg cells in the spinal cord of mice. **A**: The knockout efficiency of Treg cells was verified by immunofluorescence staining. **B** The knockout efficiency of Treg cells was verified by western blotting. **Fig. S2.** Dil-labeled exosomes are phagocytosed by bEND.3 cells. **Fig. S3.** Selection and validation of miR-2861. **A**: The intersection of 4 samples from the GEO database yielded 5 miRNAs containing miR-2861. **B** Relative expression of miR-2861 in exosome-treated bEND.3 cells (n = 3).

## Data Availability

The underlying data for this article can be found in the article and its supplementary materials.
