# Correction: Treg cells-derived exosomes promote blood-spinal cord barrier repair and motor function recovery after spinal cord injury by delivering miR-2861

**DOI:** 10.1186/s12951-023-02188-4

**Published:** 2023-12-20

**Authors:** Guang Kong, Wu Xiong, Cong Li, Chenyu Xiao, Siming Wang, Wenbo Li, Xiangjun Chen, Juan Wang, Sheng Chen, Yongjie Zhang, Jun Gu, Jin Fan, Zhengshuai Jin

**Affiliations:** 1 The Affiliated Jiangsu Shengze Hospital of Nanjing Medical University, Suzhou, Jiangsu China; 2 The First Affiliated Hospital of Nanjing Medical University, Nanjing, Jiangsu China; 3 Nanjing Medical University, Nanjing, Jiangsu China; 4 Department of human anatomy, School of Basic Medicine, Nanjing Medical University, Nanjing, Jiangsu China

**Correction: Journal of Nanobiotechnology (2023) 21:364**

10.1186/s12951-023-02089-6

Following publication of the original article [1], details for affiliations of all authors were incorrectly given as

“1 The First Affiliated Hospital of Nanjing Medical University, Nanjing, Jiangsu, China 2 Gusu School, Nanjing Medical University, Suzhou, Jiangsu, China 3 Department of human anatomy, School of Basic Medicine, Nanjing Medical University, Nanjing, Jiangsu, China 4 The Affiliated Jiangsu Shengze Hospital of Nanjing Medical University, Suzhou, Jiangsu, China”, but should have been “1 The Affiliated Jiangsu Shengze Hospital of Nanjing Medical University, Suzhou, Jiangsu, China 2 The First Affiliated Hospital of Nanjing Medical University, Nanjing, Jiangsu, China 3 Nanjing Medical University, Nanjing, Jiangsu, China 4 Department of human anatomy, School of Basic Medicine, Nanjing Medical University, Nanjing, Jiangsu, China”.

The original article [1] has been corrected.
