# Supplementary material for: Treg cells-derived exosomes promote blood-spinal cord barrier repair and motor function recovery after spinal cord injury by delivering miR-2861
Source: J Nanobiotechnology. 2023 Oct 4;21:364. doi: 10.1186/s12951-023-02089-6 (PMC10552208; doi:10.1186/s12951-023-02089-6)
Supplement: Supplementary file 1 — Additional file 1: Fig. S1. Validation of the knockout efficiency of Treg cells in the spinal cord of mice. A: The knockout efficiency of Treg cells was verified by immunofluorescence staining. B The knockout efficiency of Treg cells was verified by western blotting. Fig. S2. Dil-labeled exosomes are phagocytosed by bEND.3 cells. Fig. S3. Selection and validation of miR-2861. A: The intersection of 4 samples from the GEO database yielded 5 miRNAs containing miR-2861. B Relative expression of miR-2861 in exosome-treated bEND.3 cells (n = 3). [file 12951_2023_2089_MOESM1_ESM.docx]

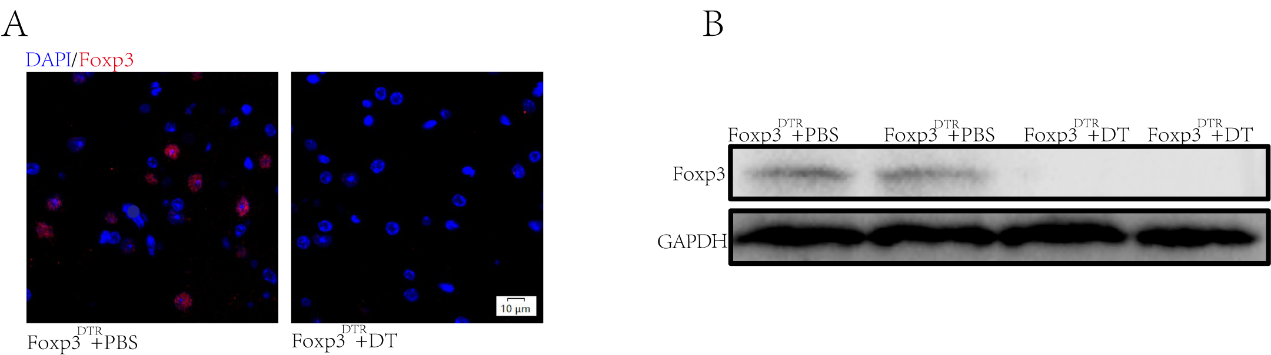
 Fig. S1: Validation of the knockout efficiency of Treg cells in the spinal cord of mice. (A): The knockout efficiency of Treg cells was verified by immunofluorescence staining. (B): The knockout efficiency of Treg cells was verified by western blotting.


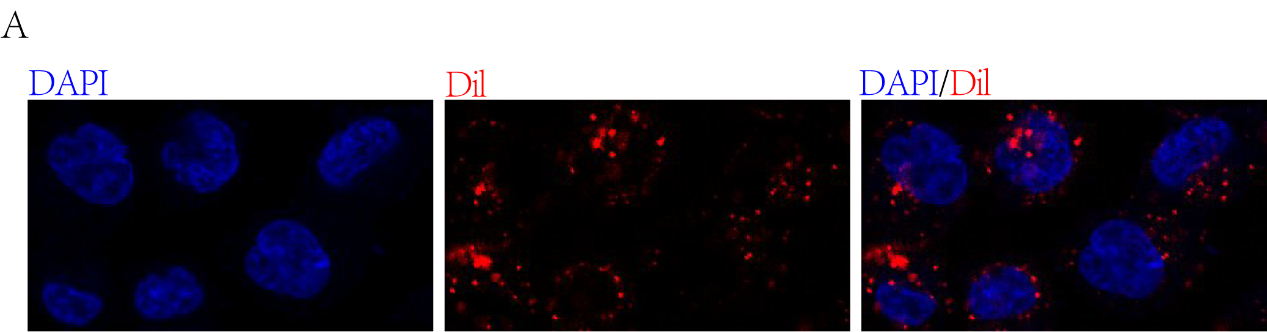
Fig. S2: Dil-labeled exosomes are phagocytosed by bEND.3 cells.


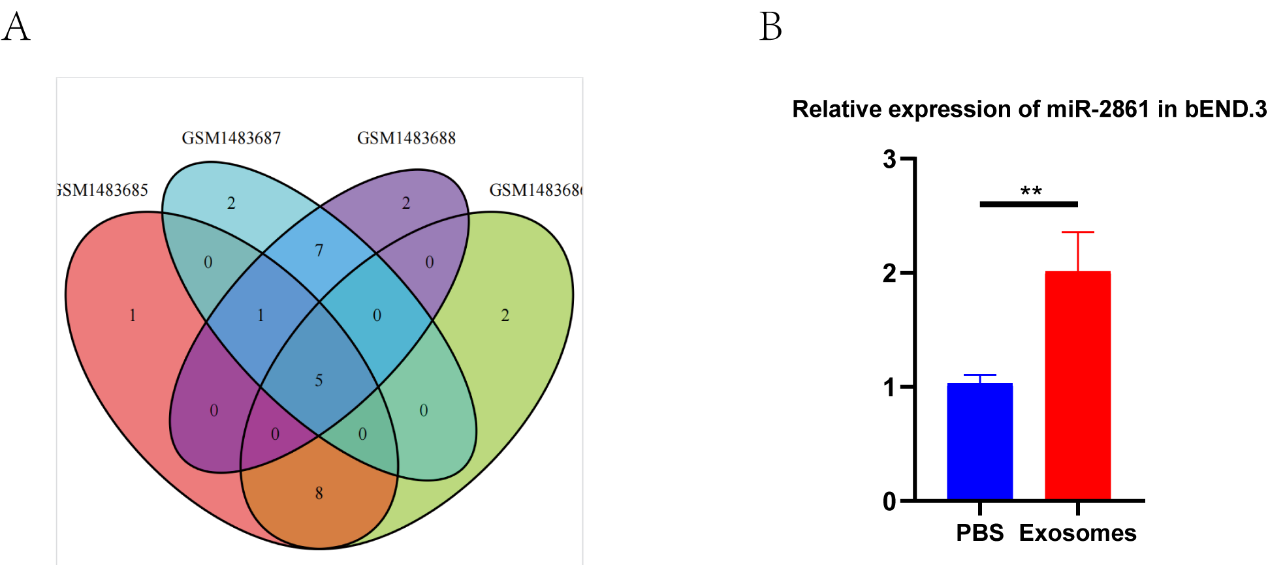
Fig. S3: Selection and validation of miR-2861. (A): The intersection of 4 samples from the GEO database yielded 5 miRNAs containing miR-2861. (B): Relative expression of miR-2861 in exosome-treated bEND.3 cells (n = 3).
